# Effectiveness of Two Plant-Based In-Feed Additives against an *Escherichia coli* F4 Oral Challenge in Weaned Piglets

**DOI:** 10.3390/ani11072024

**Published:** 2021-07-06

**Authors:** Daniel Montoya, Matilde D’Angelo, Susana M. Martín-Orúe, Agustina Rodríguez-Sorrento, Mireia Saladrigas-García, Coralie Araujo, Thibaut Chabrillat, Sylvain Kerros, Lorena Castillejos

**Affiliations:** 1 Animal Nutrition and Welfare Service (SNIBA), Animal and Food Science Department, Facultat de Veterinària, Universitat Autònoma de Barcelona, 08193 Bellaterra, Spain; dmontoya@agromel.com (D.M.); matilde.dangelo@uab.cat (M.D.); agusrodsor@gmail.com (A.R.-S.); mireia.saladrigas@uab.cat (M.S.-G.); lorena.castillejos@uab.cat (L.C.); 2 Phytosynthese, 57 Avenue Jean Jaurès, 63200 Mozac, France; coralie.araujo@phytosynthese.com (C.A.); thibaut.chabrillat@phytosynthese.com (T.C.); sylvain.kerros@phytosynthese.com (S.K.)

**Keywords:** phytogenics, piglets, ETEC F4, oral challenge, diarrhea, weaning, essential oils, Pig-MAP, *REG3G*

## Abstract

**Simple Summary:**

Phytogenic feed additives are botanic origin compounds added to animal diets with organoleptic and bioactive properties that produce benefits on performance, health, and welfare, and they contribute to reducing the use of antibiotics based on the antimicrobial properties of many of them. Globally, their use as in-feed additives in pig diets has become more frequent, especially during the weaning period. Weaning is a particularly stressful period for the young pig that is associated with an abrupt change from the mother’s milk to the dry feed and frequent outbreaks of digestive disorders and diarrhea, which is the main cause of mortality at this age. The present study aimed to evaluate the potential of two plant-based feed supplementations to improve pig adaptation to weaning and to reduce the incidence of post-weaning colibacillosis by using an experimental model of disease. Our work showed that both supplements helped piglets fight enterotoxigenic *E. coli* but probably by means of different modes of action. Whereas the supplement based on essential oils seems to improve the microbiota balance, increasing the fecal lactobacilli/coliforms ratio, the combined supplement of essential oils and non-volatile compounds seems to have anti-inflammatory properties with a reduction in the intestinal damage and an improved immune response.

**Abstract:**

This study evaluates the efficacy of two plant-based feed supplementations to fight colibacillosis in weanlings. A total of 96 piglets (32 pens) were assigned to four diets: a control diet (T1) or supplemented with ZnO (2500 ppm Zn) (T2) or two different plant supplements, T3 (1 kg/t; based on essential oils) and T4 (T3 + 1.5 kg/t based on non-volatile compounds). After one week, animals were challenged with ETEC F4, and 8 days after, one animal per pen was euthanized. Performance, clinical signs, microbial analysis, inflammatory response, intestinal morphology, and ileal gene expression were assessed. ZnO improved daily gains 4 days after challenge, T3 and T4 showing intermediate values (96, 249, 170, and 157 g/d for T1, T2, T3, and T4, *p* = 0.035). Fecal lactobacilli were higher with T3 and T4 compared to ZnO (7.55, 6.26, 8.71, and 8.27 cfu/gFM; *p* = 0.0007) and T3 increased the lactobacilli/coliforms ratio (*p* = 0.002). T4 was associated with lower levels of Pig-MAP (*p* = 0.07) and increases in villus/crypt ratio (1.49, 1.90, 1.73, and 1.84; *p* = 0.009). Moreover, T4 was associated with an upregulation of the *REG3G* gene (*p* = 0.013; *p*_FDR_ = 0.228) involved in the immune response induced by enteric pathogens. In conclusion, both plant supplements enhanced animal response in front of an ETEC F4 challenge probably based on different modes of action.

## 1. Introduction

Weaning is one of the most critical periods in the piglet life, with severe consequences on performance throughout their full productive lifetime. In commercial farms, piglets are weaned around 21–28 days of life. At this early age, weaning entails a big challenge for the pig, as they are exposed to new social partners, an abrupt dietary change, and an immature immune system [1]. As a consequence of the high stress to which pigs are subjected, a decreased feed intake is frequently observed. These circumstances are associated with a decrease in nutrients supply and their digestive capacity, a low weight gain, and a high diarrhea incidence, which could even lead to death [2]. The emergence of opportunistic pathogens during this stage, such as *Escherichia coli*, can trigger post-weaning diarrhea (PWD), which is also called post weaning enteric colibacillosis [3].

To prevent and avoid the occurrence of these series of events, antimicrobial compounds have been extensively used in the past, either as antibiotic growth promoters (AGPs) or at sub-therapeutic quantities to increase animal growth rates and to improve feed efficiency. Nevertheless, due to the strong political and social pressure to prevent antibiotic resistance in pathogenic microbiota, the supplementation of AGPs in animal feed was finally banned in the European Union, and in the most recent years, different programs have been implemented looking for a responsible use of antibiotics in livestock (European Surveillance of Veterinary Antimicrobial Consumption (ESVAC) project) [4]. This decision lessened the productivity and profitability of animal production systems [5] but also provided an opportunity to implement better zootechnical practices and fundamental research to develop new feed additives (i.e., probiotic, prebiotic, acidifiers, enzymes, phytogenic, etc.) as an alternative to AGPs in animal production [6].

Phytogenic feed additives (often called phytobiotics or botanicals) are plant-derived compounds that may have positive effects on animal growth and health due to their antibacterial, anti-inflammatory, and antioxidant properties [7,8]. In addition, their aromatic and oily characteristics have been shown to increase the palatability of feed with no flavor or aroma difference in the finished meat product [9]. Phytogenic substances utilized in phytogenic feed additives include herbs, spices, essential oils, and non-volatile extracts [10].

Studies evaluating the effects of phytogenic supplementation in swine have reported in many occasions positive results in growth performance, feed conversion ratio, and nutrient digestibility [11], amongst others. Positive results in performance have been attributed to different modes of action including direct antimicrobial effects on particular bacterial groups [12], positive effects on intestinal morphology and intestinal function [13], immunomodulatory activities in the host [14], and antioxidant properties [15]. Moreover, the effects of phytogenic compounds on maternal transfer have also been studied as an effective alternative way to improve the response of the offspring [16]. However, a closer analysis of the action of phytogenics on intestinal health and particularly on the microbial environment and the cross-talk with the host is necessary to understand how plant extracts can influence animal performance.

Thus, following the hypothesis that it is possible to improve the response of piglets to weaning and to reduce the incidence of post-weaning diarrhea by including phytogenic blends in the diet as an alternative to antimicrobials, the objective of the present study was to evaluate, in weaned piglets, the potential of two plant-based in-feed additives in front of an oral ETEC F4 challenge, analyzing their effects on performance, clinical response, immune system, and gut health. For that, we compared two diets supplemented with different phytogenic blends with a non-supplemented diet and a diet including pharmacological levels of ZnO as a positive control considering its largely known efficacy to prevent post-weaning colibacillosis [17]. 

## 2. Materials and Methods

The trial was performed at the Experimental Unit of the Universitat Autònoma de Barcelona (UAB) conducted as a Level 2–High Risk Biosecurity Procedure. It received prior approval (Permit No. CEEAH: 4026 DMAH: 10118) from the Animal and Human Experimental Ethical Committee of this Institution. The treatment, management, housing, husbandry, and slaughtering conditions conformed to European Union Guidelines [18]. All efforts were made to minimize animal suffering.

### 2.1. Animals and Housing

For the trial, 96 male piglets ((Landrace × Large White) × Pietrain) were obtained from a commercial farm, from non-vaccinated sows with *E. coli*. The piglets were weaned at 21 days of age and had an average body weight (BW) of 4.8 ± 0.62 kg. Piglets were transported to facilities of the UAB, identified, weighed, and distributed among 32 pens (3 animals per pen) within 4 rooms (8 pens per room) on arrival. Each pen was readjusted by weight to start the experiment with the same initial average weight in all pens. Each pen (3 m^2^) had a feeder and a water nipple to provide food and water ad libitum. The weaning rooms were equipped with automatic heating, forced ventilation, and an individual heat-light per pen. The experiment was conducted during the winter season (January), with an average room temperature of 28 °C ± 4 °C. 

### 2.2. Experimental Diets

Four dietary treatments were included in the study: (1) a control basal diet (T1); (2) T1 supplemented with ZnO (as ZINCOTRAX at 3100 mg/kg of feed, equivalent to 2500 ppm Zn/kg of feed) (T2); (3) T1 supplemented with plant supplements based on essential oils (*ColiFit Icaps C*, 1 kg/tm) (T3); and (4) T3 supplemented with plant supplements based on non-volatile compounds (*Phyto Ax’Cell*, 1.5 kg/tm) (T4).

All diets were given in a mash form. Basal diet (Table 1 and Table 2) was formulated to satisfy the nutrient requirement standards for pigs [19]. Basal diet was made in a single batch and subsequently divided into four batches to include the different additives to form the four experimental diets. Before being included in the mixer, the required amount of the additives was pre-mixed by hand in approximately two kg of basal diet. Chemical analyses of the diets (Table 2) including dry matter (DM), ash, gross energy, crude protein, and crude fat (diethyl ether extract) were performed according to the Association of Official Agricultural Chemists standard procedures [20]. Neutral-detergent fiber and acid-detergent fiber were determined according to the method of Van Soest et al. [21]. 

### 2.3. Plant Supplements Analyses

The tested products, *ColiFit Icaps C* and *Phyto Ax’Cell*, were two commercial products supplied and analyzed by Phytosynthese, Mozac, France. 

The product *ColiFit Icaps C* consisted of a blend of essential oils rich in trans-cinnamaldehyde, eugenol, carvacrol, thymol, and diallyl disulfure. *Phyto Ax’Cell* (Phytosynthese, Mozac, France) was based on a blend of standardized plants, plant extracts, and green propolis extract. The active compounds contained in the product were curcuminoids, carnosic derivatives, naringin flavonoids, salicylic derivatives, and artepillin-C. 

The quantification of these active compounds was performed by Phytosynthese lab with high liquid chromatography or gas chromatography according to components. Detailed description of the methodology can be found in Appendix A.

### 2.4. Bacterial Strain 

For the trial, the enterotoxigenic *Escherichia coli* (ETEC) F4 strain COLI30/14-3 used (positive for virulence factors F4ab, F4ac, LT, STb, and EAST1 and negative for K99, F6, F18, F41, STa, VT1, VT2, and EAE) was isolated from feces of a 14-week-old pig from a farm with a clinical course of colibacillosis and provided by the Veterinary Laboratory of Diagnosis of Infectious Diseases of UAB. This strain was actually used with the same purpose in previous studies of our group [22]. The oral inoculums were prepared by an overnight incubation at 37 °C in Brain Heart Infusion (BHI) with slow agitation (1× *g*) in an orbital incubator. The final inoculum was 2 × 10^8^ CFU/mL. The final culture broth was used as the oral inoculums by preparing 96 doses of 6 mL. To quantify the inoculums (CFU/mL), serial dilutions were cultured in Tryptic Soy Agar (TSA) plates (overnight, 37 °C).

### 2.5. Experimental Design, Procedure, and Sampling

Pens were allocated to the 4 treatment groups. Animals received the experimental diets over 16 d, mortality rate was registered, and no antibiotic treatment was administered to any animals in the trials. After the adaptation period (experimental days 0 to 7), all animals were orally challenged with the pathogen ETEC F4 strain COLI30/14-3 as a single dose (1 × 10^9^ CFU). In order to ensure that the stomach was full at the time of inoculation and thus facilitate bacterial colonization, feed withdrawal was performed at 21:00 h of the previous day and provided back 30 min before inoculation.

One animal of each pen (8 per treatment) was euthanized on day 8 post-inoculation (PI). Feed intake by pen was registered at day 0, 7, 8, 9, 10, 11, 14, and 15, and individual weight was measured on day 0, 4, and 8 PI. The average daily gain (ADG), average daily feed intake (ADFI), and gain–feed ratio (G:F) were calculated for each pen. 

After the ETEC F4 challenge, animals were checked daily for clinical signs to evaluate their PI status (i.e., dehydration, apathy, and diarrhea). Fecal consistency was registered on day 0, 1, 2, 3, 4, and 7 PI; score consistency was measured using a scale from 1 to 4: 1 = normally shaped feces, 2 = shapeless soft feces, 3 = thin or liquid feces, and 4 = very liquid feces (translucid) or with blood. Rectal temperature was assessed before the challenge (d 0 PI) and day 1 and 2 PI with a digital thermometer. Blood samples were taken on day 4 and 8 PI from the initial intermediate BW piglet from each pen. For microbiological analysis, fecal samples were taken aseptically before inoculation at day 0, and at days 4 and 8 PI. Samples were always collected from the same piglet from each pen, corresponding to that with intermediate BW the first day of the trial. Fecal samples were collected after spontaneous defecation associated with manipulation of the animal or by rectal stimulation. Samples were first stored in ice until analysis. 

On day 8 PI, the pig of medium weight of each pen on arrival (N = 32) was euthanized. Sampling took place during the morning (between 8:00 and 14:00). Prior to euthanasia, a 10 mL sample of blood was obtained by venipuncture of the cranial vena cava using 10 mL tubes without anticoagulant (Aquisel; Madrid, Spain), and immediately after sampling, piglets received an intravenous, lethal injection of sodium pentobarbital in the same vein (200 mg/kg BW; Dolethal, Vetoquinol S. A.; Madrid, Spain). Once dead, the animals were bled, their abdomens were immediately opened, and the whole gastrointestinal tract was excised. Digesta (approximately 40 mL) from the ileum and proximal colon (considered to be 0.75 m from the ileocecal junction) was homogenized, and samples were collected for bacterial counts. The pH of the homogenized digesta content was immediately determined with a pH-meter (Crison 52-32 electrode, Net Interlab; Barcelona, Spain). For histological study, 3 cm sections were taken from the proximal ileum and proximal colon, were opened longitudinally, washed thoroughly with sterile phosphate-buffered saline (PBS), and fixed by immersion in a formaldehyde solution (4%). Blood samples were centrifuged (3000× *g* for 15 min at 4 °C) to obtain serum, and the serum obtained was divided into different aliquots and stored at −80 °C. For intestinal gene expression analysis, 3 cm long sections were removed from the mid jejunum, opened longitudinally, and washed exhaustively with sterile PBS. A small piece of fresh tissue was cut at around 0.5 cm^2^ and placed in 5–10 volumes of RNAlater solution. Samples were stored at 4 °C overnight (to allow the solution to penetrate the tissue) and then moved to −80 °C until RNA isolation. For analyzing enterobacteria, coliforms and *E. coli* F4 attached to the ileal mucosa, 5 cm long sections of ileum were collected from each animal, washed thoroughly three times with sterile PBS, opened longitudinally, and scraped with a microscopy glass slide to obtain the mucosa scraping contents. One aliquot of each scraping sample was kept on ice until traditional microbiological analysis, and a second aliquot was kept on dry ice and subsequently in the freezer (−80 °C) until DNA analysis. 

### 2.6. Analytical Procedures

For microbial counts of enterobacteria and total coliforms, the ileum and colon contents, ileum scrapings, and feces were suspended in PBS (1:10) and homogenized for 5 min. Thereafter, 10-fold serial dilutions were made in PBS to seed in chromogenic and Rogosa agar for *E. coli* and lactobacilli counting, respectively. Counts were read after 24 h of incubation at 37 °C. 

For morphological measures, tissue samples were dehydrated and embedded in paraffin wax, sectioned in 4 µm thick slices, and stained with hematoxylin and eosin. Morphological measurements of ileal sections were performed with a light microscope (BHS, Olympus) using the technique described in Nofrarías et al. [23]. Measured parameters included villus height, crypt depth, villus/crypt ratio, intraepithelial lymphocytes (IEL), goblet cells (GC), and mitosis. 

Serum concentrations of tumor-necrosis factor-α (TNF-α) were determined using Quantikine Porcine TNF-α kits (R&D Systems). The Pig major acute-phase protein (Pig-MAP) concentration was determined using a sandwich-type ELISA (Pig MAP Kit ELISA, Pig CHAMP Pro Europe S.A.), according to the manufacturer’s instructions.

Total RNA was extracted from 50 mg of mid jejunum tissue through homogenization with a Polytron device (IKA, Staufen, Germany) in 1 mL of TRIzol reagent (Thermo Fisher Scientific, Waltham, MA, USA), followed by of the use of the Ambion RiboPure Kit (Thermo Fisher Scientific, Waltham, MA, USA) according to the manufacturer’s protocol. The quantification and purity valuation of the extracted RNA was assessed by measuring the absorbance at 230, 260, and 280 nm using a NanoDrop ND-1000 spectrophotometer (NanoDrop Technologies Inc., Wilmington, DE, USA). The RNA integrity was checked with Agilent Bioanalyzer-2100 equipment (Agilent Technologies, Santa Clara, CA, USA), following the producer’s protocol. 

For cDNA synthesis, 1 μg of total RNA was reverse-transcribed into cDNA in a final volume of 20 µL. A High-Capacity cDNA Reverse Transcription Kit (Applied Biosystems, Foster City, CA, USA) and random primers were used, and the following thermal profile was applied: 25 °C for 10 min; 37 °C for 120 min; 85 °C for 5 min; 4 °C hold. cDNA samples were stored at −20 °C until use. 

Intestinal gene expression analysis was made using a custom OpenArray plate. A total of 56 genes (including 4 house-keeping genes: *ACTB*, *B2M*, *GAPDH*, and *TBP*) related to intestinal health were selected, as described by Reyes-Camacho et al. [16] (Table A1). The description of forward and reverse primers for each gene is found in Table A2. One replicate per sample was run in a Taqman Open Array gene expression custom plate format for gene expression with 56 assays of 48 samples per plate (OpenArray plate) in a QuantStudio 12K Flex Real-Time PCR System (Applied Biosystems, Foster City, CA, USA). Gene expression data were analyzed using the ThermoFisher Cloud software 1.0 (Applied Biosystems, Foster City, CA, USA) applying the 2^−ΔΔCt^ method for relative quantification (RQ) and using the sample with the lowest expression as a calibrator.

### 2.7. Statistical Analyses

Microbiological counts were log transformed for analysis. The general linear and/or the mixed models of SAS version 9.2 (SAS Institute Inc., Cary, NC, USA) were used to analyze the effect of experimental treatments on different parameters except gene expression (see below). The diet was considered a fixed effect, and random effect was used to account for variation between pens. When treatment effects were established, treatment means were separated using the probability of differences function adjusted by Tukey–Kramer. The pen was considered the experimental unit. The α-level used for the determination of significance was *p* = 0.05. The statistical trend was considered for *p* < 0.10.

For gene expression statistical analysis, the open-source software R v3.5.3. was used. The RQ data matrix was used and normalized accordingly to the reference genes. Genes with normal distributions were analyzed using One-Way ANOVA, while genes with non-normal distributions were analyzed using the non-parametric Kruskal–Wallis test. *p*-values were adjusted using the False Discovery Rate (FDR) method, and Tukey tests were performed for genes with significant differences between treatments.

## 3. Results

Animals showed a good health status at the beginning of the experiment, and following the oral challenge with ETEC, animals developed moderate clinical signs of diarrhea that began to resolve spontaneously at the end of the study. No antibiotic nor pharmacological treatment was administered to any of the animals during the trial. A total of six casualties were registered in the days following inoculation that were attributed to post-weaning stress and subsequent bacterial challenge. Specifically, there were three deaths in the T1 group (on day 4 PI), two in the T4 group (one on day 3 PI and another one on day 4 PI), and one in the T3 group (on day 4 PI). All of these animals had previously shown symptoms of apathy. 

Due to inexplicable reasons, in three pens, animals registered an extremely low feed intake (72, 52, and 49 g for each pen (three animals) throughout the whole experiment). Considering that these animals had not received the in-feed treatments, these replicates were removed from the study. Removed pens belonged to T3 (two pens) and to T4 (one pen) treatments.

### 3.1. Plant Extract Composition

The main active compounds contained in *ColiFit Icaps C* and *Phyto Ax’Cell* are shown in Table 3. The concentrations (mg/kg) of trans-cinnamaldehyde, eugenol carvacrol, thymol, and diallyl disulfure are shown for *ColiFit Icaps C* and equivalent in the T3 and T4 diets (1 kg/t level of inclusion). The concentrations (mg/kg) of curcuminoids, carnosic derivatives, salicylic derivatives, flavonoids (naringin), and artepilin-C are shown for *Phyto Ax’Cell* and equivalent in the T4 diet (1.5 Kg/t level of inclusion). 

### 3.2. Animal Performance

The effects of the experimental treatments on the evolution of body weight (BW), average daily feed intake (ADFI), average daily gain (ADG), and gain:feed ratio (G:F) throughout the trial are shown in Table 4. No statistical differences related to the experimental treatments were found in the final body weight of the animals at the end of the study.

No significant differences were found in ADFI despite numerical increases observed in the animals receiving ZnO (T2) or phytogenics (T3 and T4). The ADG showed a significant increase after the challenge (0–4 PI period) for the animals receiving the ZnO supplementation (*p* = 0.035), while pigs receiving phytogenics showed intermediate values. The impact of the supplemented diets in ADG was also reflected in the gain/feed ratio (G:F) (0–4 PI period) that also showed the highest values with ZnO compared to control (*p* = 0.039) and intermediate values for T3 and T4.

**Figure 1:**
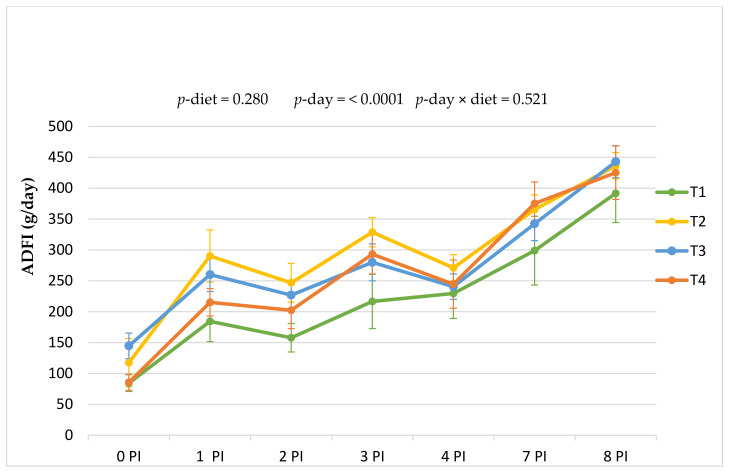
Effect of the experimental treatments on the average daily feed intake of weaning piglets orally challenged with ETEC F4. The line chart shows the evolution along the post-inoculation period (days 0 to 8 PI). Treatments: T1, basal diet; T2, basal diet + ZnO; T3, basal diet + plant supplement *ColiFit Icaps C*; T4, T3 + plant supplement *Phyto Ax’Cell*.

After the challenge, feed intake was also registered in a daily pattern. Figure 1 shows ADFI evolution. As expected, feed intake increased significantly with time (*p* day < 0.0001), although no differences were found between dietary treatments (*p* = 0.280) nor interaction (*p* = 0.521). Despite the lack of statistical differences, it is fair to highlight that mean values for animals receiving the basal diet (T1) were the lowest throughout the trial, and animals receiving ZnO (T2) exhibited the highest values from day 1 to 3 PI.

### 3.3. Intestinal Microbiota

Table 5 shows the microbial counts of lactobacilli and total coliforms for the different samples collected during the assay. No significant differences in total coliforms between treatments were detected in fecal samples, ileal scrapings, nor ileal or colon digesta. However, on day 8 PI, the numbers of lactobacilli counts in feces were the lowest with the diet supplemented with ZnO and the highest with diets supplemented with plant supplements (T3 & T4), the difference being more than 2 log units (*p*-diet = 0.0007). A decrease in the lactobacilli population with the supplementation of ZnO was also observed in colon digesta on day 8 PI (*p*-diet = 0.0336).

Microbiological results were also expressed as the lactobacilli/coliforms ratio. No differences were found in ileal scrapings nor ileal or colon digesta samples between diets at day 8 PI. However, in fecal samples, significant differences were found at day 8 PI (*p* = 0.002), with the T3 diet showing higher values than the basal diet and the ZnO supplemented diet. The T4 diet showed intermediate values.

### 3.4. Clinical Signs

**Figure 2:**
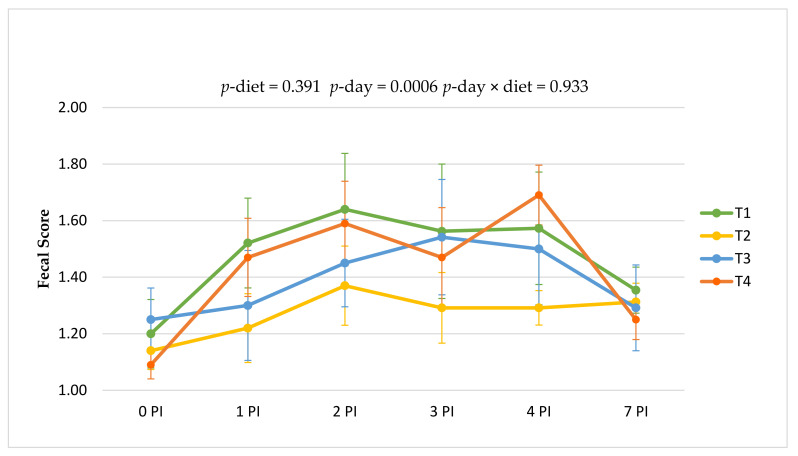
Effects of the experimental treatments on fecal consistency during the post-challenge period. Experimental days 8 to 15 (0 to 7 PI). Treatments: T1, basal diet; T2, basal diet + ZnO; T3, basal diet + plant supplement ColiFit Icaps C; T4, T3 + plant supplement *Phyto Ax’Cell*. Fecal consistency was measured using a scale from 1 to 4: 1 = normally shaped feces, 2 = shapeless soft faces, 3 = thin or liquid faces and 4 = very liquid faces (translucid) or with blood.

Figure 2 shows the fecal consistency evolution throughout the post-inoculation period. The oral challenge promoted a mild course of diarrhea that was translated into a significant increase in fecal scores after the challenge (*p*-day = 0.0006). Differences in fecal consistency due to treatments were not significant (*p* = 0.391), and neither was the interaction with time (*p* = 0.933). However, it should be underlined that animals receiving the ZnO treatment (T2) always showed the lowest scores in the 1–4 PI period.

Rectal temperatures before inoculation (on day 0 PI (38.8 ± 0.11)), at 24 and 48 h post-challenge (on day 1 PI (38.9 ± 0.09) and on day 2 PI (39.0 ± 0.12), respectively) were in all cases within the physiological range, and no differences were seen between treatments. 

pH Values of ileum (6.56 ± 0.041) and colon digesta (6.14 ± 0.073) were registered on day 8 PI, and no differences were seen between treatments in any of the segments of the gastrointestinal tract.

### 3.5. Inflammatory Response

Serum concentration of Pig major acute-phase protein (Pig-MAP) and tumor-necrosis factor-α (TNF-α) were determined after *E. coli* challenge (Table 6). At day 4 PI, a trend for Pig-MAP to decrease with T4 (*p* = 0.070) was found; T4 was the only treatment that showed mean values of Pig-MAP below 2 mg/mL, which was reported as a reference value for inflammatory response [24]. No significant changes were detected in TNF-α, except a numerical trend with ZnO to decrease at day 4 PI (*p* = 0.124).

### 3.6. Intestinal Morphology

The changes induced by the experimental diets regarding histomorphological parameters at day 8 PI are shown in Table 7. The supplementation with ZnO (T2) and the T4 treatment significantly improved the villus/crypt ratio compared to the plain basal diet (T1) (*p* diet = 0.009). The T3 diet showed intermediate values. On the other hand, villus height was not modified by experimental diets, neither were the crypt depth nor the villus intraepithelial lymphocytes, nor the villus goblet cells or mitosis. 

### 3.7. Jejunal Gene Expression

**Figure 3:**
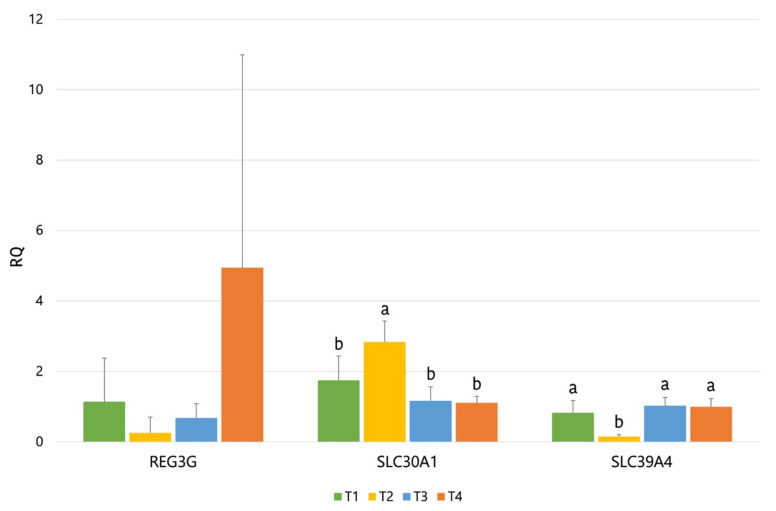
Effects of experimental treatments on jejunal gene expression of piglets orally challenged with ETEC F4. Only genes with *p_FDR_* < 0.25 are included. Values are expressed as relative fold changes (RQ) normalized by housekeeping genes *GAPDH*, *ACTB*, *TBP*, and *B2M*. Bars with different letters within a gen mean the differences between treatments were *p* < 0.02. Treatment means with different letters within a gen are statistical different (*p* < 0.05).

From a total of 56 quantified genes, only *SLC30A1* (Solute carrier family 30 (zinc transporter) member 1) and *SLC39A4* (Solute carrier family 39 (zinc transporter) member 4) were shown to be significantly modified by the experimental treatments (see Figure 3). The *SLC30A1* was shown to be upregulated by ZnO supplementation, and *SLC39A4* was downregulated (T1 vs. T2, *p* < 0.004) (see Table 8). Both genes codify for solute carrier proteins involved in the cellular transport of Zn. Additionally, the *REG3G* gen (Figure 3) also showed a numerical trend to be upregulated by the T4 diet (*p_FDR_* < 0.23). The *REG3G* gen (regenerating islet-derived protein 3 gamma) codifies for an antimicrobial peptide produced by the Paneth cells via stimulation of Toll-like receptors (TLRs) by pathogen-associated molecular patterns (PAMPs).

## 4. Discussion

The present study aimed to evaluate the efficacy of two plant-based feed supplementations in comparison to ZnO to fight colibacillosis in weaned piglets by using an experimental model of disease. To assess this objective, two different phytogenic additives containing extracts and essential oils from plants were tested in this trial; T3 included a blend of different essential oils while T4 contains in addition non-volatile compounds with putative antioxidant, anti-inflammatory, and immunomodulatory properties. 

In general terms, performance was not significantly affected by the dietary treatments compared to the plain diet before or after the challenge. The limited number of replicates in this kind of controlled-challenge trials probably precluded us to detect significant differences in performance. Despite this, some numerical trends were detected for treated groups with ZnO or plant supplements. 

Regarding the impact of phytogenics on feed intake, results found in the literature on ADFI in pigs are variable. In the present study, the dietary inclusion of the two botanic feed supplements did not significantly affect the overall ADFI intake, which is in line with other studies in weaned piglets [8,25,26,27]. However, Platel and Srinivasan [28] described how many botanical compounds and spices can improve food intake in humans and other mammal species. A digestive stimulant action mediated by an increase of salivary, gastric, or bile secretions, and reduction in food transit time, may result in a stimulation of appetite. In this regard, Cho et al. [29], Kommera et al. [30], and Maenner et al. [31] reported an enhanced ADFI with the use of phytogenics containing fenugreek, clove, cinnamon; anis oil, citrus oil, oregano oil, natural flavor; and menthol and cinnamon aldehyde, respectively. In contrast to this, other authors observed a decrease of the ADFI in weaned piglets due to the supplementation of cinnamon, thyme, oregano; and buckwheat, thyme, curcuma, black pepper, and ginger, respectively [32,33]. Therefore, the results found in the literature are controversial and probably respond to different products, blends, and doses tested. 

Regarding ADG, in this work, we did not find statistical differences between treatments in the adaptation week, but differences were found in the period immediately after the challenge (0–4 PI period; *p* = 0.035)). Four days after the challenge, the T2 ADG (including ZnO) was improved significantly compared to the basal diet T1, while animals receiving plant blends (T3 and T4) showed intermediate values. These results are in concordance with other studies that evaluated the effect of the addition of essential oils [8] and phytogenic products based on plant extracts plus essential oils as anise oil, clove oil, citrus oil, and oregano oil, amongst others [23,30,31,34]. In contrast, Namkung et al. [32] described a decrease of the ADG in weaned piglets, which were offered a diet with a herbal extract containing cinnamon, thyme, and oregano. Other authors have not found differences in weaned piglets fed diets supplemented with different essential oil blends [35,36], which is concurrent with other nursery pig studies involving phytogenic feed additives in which no effect on ADG and/or BW and/or ADFI were reported [9,26,37,38].

The increase of ADG in the supplemented diets was also reflected in the significant increment of gain/feed ratio (G:F) during the 0–4 PI period in the piglets receiving the T2 diet compared to T1, while plant-supplemented diets (T3 and T4) showed again intermediate values. In this respect, Kommera et al. [30] also reported an improvement of the G:F in weaned piglets that were offered diets containing anise, citrus, and oregano oils. In contrast, Namkung et al. [32] attributed negative feed efficiencies found for piglets during the first post-weaning week to the strong smell of cinnamon, thyme, and oregano, which was consistent with the findings of other works that also reported lower G:F in groups of piglets offered diet supplemented with essential oils (cinnamaldehyde and thymol being the main active components) [8,39] or anise [40].

In our study, increases in ADG and G:F observed with T2 and numerically with T3 and T4 were only found in the period immediately post-challenge but not the week before and neither in the 4–8 PI period. Therefore, these changes can be interpreted as an improvement of the animal response to the pathogen challenge. As we see, there is a wide variability on the performance results reported among authors due to the supplementation of phytogenic compounds in the swine diets. Clouard and Val-Laillet [27] suggested that several factors, such as animal characteristics (i.e., age, sex, physiological status), experimental conditions, time of exposure and particularly dosage, and biochemical features of phytogenic might be decisive factors in the development of pig performance. In addition, the diversity of plant materials and the lack of description of tested substances available in the literature make it difficult to compare these results with other phytogenics.

Post-weaning diarrhea is one of the many interdependent factors causing the high mortality rate in piglets. The addition of phytogenic feed additives has been reported to control clinical diarrhea [8,39,41]. The reasons for such improvement are most likely associated with the reduction of *E. coli* load in the gut, especially when piglets are raised under relatively poor environmental conditions. Lee et al. [42] reported that both thymol and cinnamaldehyde have antimicrobial and anti-inflammatory effects. Different studies report that different combinations of essential oils exhibit antimicrobial in vitro activity against potentially pathogenic bacteria such as *E. coli*, *Clostridium perfringens*, *Salmonella*, *Listeria*, or *Staphylococcus*, while they do not inhibit the growth or had less activity against beneficial microbes such as *Enterococcus*, *Bacillus*, *Lactobacillus*, and *Bifidobacterium* [43,44]. Specifically, a study made by Girard et al. [45] with the same plant supplement as diet T3 (*ColiFit Icaps C*) was able to inhibit *E. coli* growth even at sub-MIC (minimum inhibitory concentration) concentrations with a bacteriostatic action. This effect was correlated to a reduction of membrane permeability of *E. coli* with a significant degradation of their membrane polarity [46]. Moreover Kerros et al. [47], also with this essential oil mix, evidenced MICs on *Lactobacillus* species equal or higher than those determined for pathogens *E. coli* and *Clostridium jejuni*, providing evidence of the selectivity of this phytogenic. According to this observation, in the present study, the numbers of lactobacilli in feces and colon digesta on day 8 PI were the highest with diets including the plant additives (T3 and T4) and the lowest with the T2 treatment. The lactobacilli/coliforms ratio, as a potential index of the microbiota balance, was also found to be increased at day 8 PI in feces by the T3 treatment when compared to the basal diet, showing diet supplemented with T4 intermediate values. However, in our study, we were not able to demonstrate a significant impact of the treatments on incidence of diarrhea prevalence or fecal score. Other authors also have failed to demonstrate significant improvement in these parameters supplementing phytogenic mixtures [25,26,29,30,48,49]. However, we cannot discard that limitations in the experimental models of disease and in the number of replicates would have precluded us from identifying significant changes.

The inclusion of phytogenic in the current diets did not affect the serological concentrations of cytokine TNF-α but showed a trend in the major acute-phase protein Pig-MAP to decrease at day 4 PI with T4 (*p* = 0.07). These results would suggest that this phytogenic blend could exhibit anti-inflammatory properties in front of the tissue injury promoted by the ETEC challenge, since in swine, Pig-MAP have been related to acute inflammatory processes and also to the extent of tissue injury [24]. In addition, recent study has demonstrated the usefulness of this biomarker to determine the intestinal injury degree and barrier integrity in recently weaned pigs subjected to an ETEC oral challenge [50]. To appreciate this result, other authors have described how phytogenic agents could exert immune-modulating properties. In this regard, Machado et al. [51] demonstrated that two Brazilian Green Propolis extracts containing p-coumaric acid, Artepillin-C, and other minor compounds could exert a strong local and systemic anti-inflammatory action from an immunomodulatory action on pro-inflammatory (IL-6 and TNF-*α*) and anti-inflammatory (TGF-*β* and IL-10) cytokines. Likewise, Hori et al. [52] demonstrated that a Propolis Standardized Extract (EPP-AF), including caffeic, p-coumaric, trans-cinnamic acids, aromadendrin, and artepillin C could reduce the IL-1β secretion in mouse macrophages. 

The supplementation with ZnO (T2) had a positive effect on the intestinal architecture, particularly for the villus/crypt ratio. In addition, we also found significant improvements with a T4 diet, and a trend with T3. These results agree with those from other studies conducted by Zeng et al. [8] and Li et al. [39], in which dietary supplementation of essential oils improved the villus/crypt ratio in the jejunum of weaned piglets. Particularly, the treatment T4, which showed a higher impact, could have prevented the intestinal damage probably mediated by a better controlled inflammatory response according to the reduction also observed in Pig-MAP values. It is important to remark these improvements in the epithelial integrity shortly after weaning, as they could be regarded as a very positive indicator of the potential of the tested supplements to prevent post-weaning diarrhea. After weaning, a transient reduction is produced in villus height as well as an increase in crypt depth [53], which make animals more susceptible to pathogen infection and also decrease the absorptive capacity of the intestine with a reduction in feed efficiency [54]. Intestinal morphology improvement has been observed by Maneewan et al. [55] using turmeric in nursery pigs. Curcuminoids from turmeric may play a positive role on intestinal IL-1β [56] correlated with a favorable effect on tight junctions in a CaCO_2_ cell model [57]. 

Concerning gene expression, our results showed an upregulation in *SLC30A1* by ZnO supplementation and a downregulation for *SLC39A4*. Both *SLC30A1* and *SLC39A4* codify for solute carrier proteins, which are involved in the cellular transport of Zn. On the one hand, *SLC30A1* (Solute Carrier Family 30 Member 1) is involved in the transport of intracellular zinc into the extracellular matrix, and it has been shown to be upregulated by high dietary ZnO [58]. On the other hand, *SLC39A4* (Solute Carrier Family 39 Member 4) is involved in the Zn uptake from the gut lumen and has been shown to be downregulated by high dietary ZnO in piglets [58] and particularly in piglets challenged with ETEC [59]. Therefore, these results are according to what would be expected from a high supplementation of ZnO (3100 ppm). The *REG3G* gene also showed a numerical trend of being upregulated by the T4 diet. C-type lectins of the *REG3* family function as a barrier to protect body surfaces against microorganisms, modulating host defense process via bactericidal activity. A wide range of studies indicate that the *REG3G* family plays an important role in the physical segregation of microbiota from the host as well as in the immune response induced by enteric pathogens [60]. Moreover, this gene has been reported to be upregulated by ETEC challenge [61] and also by dietary prebiotics (in mice) [62]. Particularly, these last authors associated the increased expression of *REG3G* to the changes observed in the microbiota with the prebiotic that could have counteracted the inflammation induced by high-fat diets. In the present study, it could be hypothesized that the increase observed in the *REG3G* expression could be correlated to the pathogen control, to the intestine preserved from damages induced by the ETEC challenge (higher villus/crypt ratio), and to the lower expression of Pig-MAP (*p* < 0.07) observed with the T4 diet.

The results of this study evidence that plant supplements can exert positive effects in front of an ETEC F4 challenge. However, it is difficult to elucidate which specific modes of action could had been involved, and moreover which active components of these blends would be primary responsible. Complex interactions between the microbiota and the host probably are behind some of these effects, together with a direct impact of these active compounds on the host mediated by their known antioxidant and antinflammatory properties. Further research is needed to fully understand the high potential of phytogenics to improve animal health and particularly to improve the adaptation of young piglets to weaning.

## 5. Conclusions

In summary, the results of this study would suggest that both tested plant supplements could help the piglet to fight the ETEC challenges commonly faced after weaning. This efficacy would be supported by the numerical increases observed in ADG and gain/feed ratio in the period immediately after the challenge, with intermediate figures between the plain diet and the diet including pharmacological doses of ZnO. In the case of the first supplement, based on essential oils, the better response could be due to an improved microbiota balance suggested by the increased lactobacilli/coliforms ratio in feces registered at day 8 PI when compared to plain diet. Regarding the combined supplement of essential oils and non-volatile compounds, it appears to modulate the inflammatory response with lower Pig-MAP values and improved intestinal architecture with higher VH:CD ratios. These changes would also be consistent with the trend for an upregulation of the *REG3G* gene observed for this treatment (*p* = 0.013; *p*_FDR_ = 0.228). The findings of this study would support the usefulness of both plant additives during the weaning period to reduce the prevalence and severity of post-weaning colibacillosis in the pig.

## Figures and Tables

**Table 1:** Ingredient composition of the diets (as feed).

Ingredient Composition of Basal Diet (g/kg)	
Maize	207.3
Wheat	180.0
Barley 2 row	170.0
Extruded soybean	149.0
Sweet whey powder (cattle)	100.0
Soybean meal 47	80.0
Fishmeal MT	60.0
Whey powder 50% fat	25.0
Monocalcium phosphate	6.8
Calcium carbonate (CaCO_3_)	3.9
L-Lysine HCL (78)	4.5
Vit-Min Gplus *	4.0
DL-Methionine 99	2.6
Sodium chloride	2.5
L-Threonine	2.3
L-Valine	1.5
L-Tryptophan	0.6

* Provided per kilogram of complete diet: 510.0 mg butylhydroxytoluene (BHT); 3750.0 mg of Mn (glycine manganese chelate, hydrated); 15,000.1 mg of Fe (chelate glycine irons); 6250.0 mg of Zn (zinc glycine glycine chelate, solid); 20.0 mg of L-selenometionine; 5000.1 mg of Cu (copper chelate (II) of soluble glycine hydrate); 27,500.0 mg of Cu (copper sulfate II pentahydrate); 17,500.3 mg of Zn (zinc oxide); 6250.2 mg of Mn (manganese oxide); 175.0 mg of I (iodine calcium anhydrous); 50.0 mg of Se (sodium selenite); 500.1 mg of vitamin K3; 1750.4 mg of vitamin B2; 749.7 mg of vitamin B1; 1875.0 mcg of vitamin D; 9098.8 UI of vitamin E; 3,000,000.0 UI of vitamin A; 975.0 UI of vitamin E/acetate; 3750.0 UI of vitamin E/acetate; 1833.5 mg of vitamin B6; 15.0 mg of vitamin B12; 11,248.8 mg of nicotinic acid; 4250.0 mg of pantothenic acid; 50.0 mg of biotin; 374.8 mg of folic acid; 350,000.0 UI of vitamin D.

**Table 2:** Chemical analyzed composition of the diets (as feed).

Component	Analyzed Composition (g/kg)			
	Experimental Diets ^A^			
	T1	T2	T3	T4
Dry Matter	913.9	915.8	914.2	916.5
Ash	54.7	56.9	56.0	52.0
Crude fat	59.0	57.3	55.0	55.0
Crude protein	208.5	200.2	203.4	203.5
Neutral detergent fiber	147.7	139.7	138.8	129.7
Acid-detergent fiber	42.0	46.4	41.9	41.8

^A^ Treatments: T1, basal diet; T2, basal diet + ZnO; T3, basal diet + plant supplement *ColiFit Icaps C*; T4, T3 + plant supplement *Phyto Ax’Cell*.

**Table 3:** Chemical analysis of the main active compounds of the tested plant-based in-feed additives: *ColiFit Icaps C* and *Phyto Ax’Cell.* Results as gives as mg/kg of product and also as final concentrations in experimental diets.

*ColiFit Icaps C*			*Phyto Ax’Cell*		
Main Active Compounds (mg/kg)	Product	Diet (T3 and T4)	Main Active Compounds (mg/kg)	Product	Diet (T4)
Trans-cinnamaldehyde	101,218	101	Curcuminoids	3,290	4.9
Eugenol	12,400	12	Carnosic Derivatives	5,010	7.5
Carvacrol	6514	65	Salicylic Derivatives	6360	9.5
Thymol	4359	44	Naringin	966	1.5
Diallyl disulfure	1123	11	Artepilin-C	180	0.27

**Table 4:** Effect of the experimental treatments on growth performance of weaning piglets orally challenged with ETEC F4.

-	Treatments ^A^				-	-
	T1	T2	T3	T4	RSD ^B^	*p*-Value
BW ^C^ (kg)	-	-	-	-	-	-
Initial	4.85	4.85	4.87	4.58	0.062	0.959
Final	7.10	7.36	7.26	7.15	0.844	0.931
ADFI ^D^ (g/d)	-	-	-	-	-	-
Adap ^E^	84.4	117.5	144.7	85.6	59.13	0.217
0–4 PI ^F^	197.1	284.1	251.8	238.7	74.51	0.162
4–8 PI ^G^	391.7	436.2	442.9	425.1	110.83	0.813
0–8 PI ^H^	259.6	333.6	309.6	313.2	86.82	0.391
Overall ^I^	177.8	232.8	232.7	207.0	63.56	0.302
ADG ^J^ (g/d)	-	-	-	-	-	-
Adap	28.8	52.4	21.8	33.6	37.99	0.466
0–4 PI	96.3 ^b^	249.2 ^a^	170.4 ^ab^	156.9 ^ab^	97.28	0.035
4–8 PI	341.6	286.7	358.6	308.7	115.20	0.646
0–8 PI	219.0	268.0	264.5	232.8	93.55	0.689
Overall	130.2	167.4	151.2	139.8	58.86	0.632
G:F ^K^ (g/d)	-	-	-	-	-	-
Adap	0.08	0.48	0.15	0.39	0.462	0.300
0–4 PI	0.33 ^b^	0.88 ^a^	0.70 ^ab^	0.58 ^ab^	0.359	0.039
4–8 PI	1.09	0.75	0.99	0.79	0.255	0.045
0–8 PI	0.79	0.80	0.87	0.72	0.196	0.587
Overall	0.67	0.72	0.67	0.66	0.184	0.897

^A^ Treatments: T1, basal diet; T2, basal diet + ZnO; T3, basal diet + plant supplement *ColiFit Icaps C*; T3 + plant supplement *Phyto Ax’Cell*. ^B^ RSD: residual standard deviation. ^C^ BW: body weight. ^D^ ADFI: average daily feed intake. ^E^ Adap: experimental days 0 to 7. ^F^ 0–4 PI: post-inoculation period from days 0 to 4, experimental days 8 to 11. ^G^ 4–8 PI: post-inoculation period days 5 to 8, experimental days 12 to 16. ^H^ 0–8 PI: post inoculation period days 0 to 8, experimental days 8 to 16. ^I^ Overall: experimental days 0 to 16. ^J^ Average Daily Gain. ^K^ Gain/feed ratio. ^a,b^ Values with different letters within a row indicate a significant difference at *p* ≤ 0.05.

**Table 5:** Effects of the experimental treatments on plate counts of total coliforms and lactobacilli (log cfu/g fresh matter (FM)) and lactobacilli/total coliforms ratio of feces, ileal scrapings, and ileal and colonic digesta samples of weaning piglets orally challenged with ETEC F4.

-	Treatments ^A^				-	
	T1	T2	T3	T4	RSD ^B^	*p*-Value
Total Coliforms (cfu/gFM)						
Feces						
Arrival	8.04	8.04	8.61	7.11	0.939	0.072
Day 0 PI	7.66	7.44	6.79	7.39	1.337	0.677
Day 4 PI	7.90	8.43	8.28	8.33	0.896	0.673
Day 8 PI	8.59	7.91	7.27	8.20	1.106	0.187
Ileal scrapings						
Day 8 PI	5.19	5.17	5.10	6.17	1.396	0.440
Ileal digesta						
Day 8 PI	7.88	7.43	8.43	8.55	0.947	0.124
Colon digesta						
Day 8 PI	8.15	6.69	7.51	7.25	1.689	0.396
Total lactobacillus (cfu/gFM)						
Feces						
Arrival	7.11	7.26	7.18	6.92	0.748	0.879
Day 0 PI	8.76	8.78	8.53	8.84	0.528	0.747
Day 4 PI	8.22	7.37	7.27	8.05	1.252	0.389
Day 8 PI	7.55 ^ab^	6.26 ^b^	8.71 ^a^	8.27 ^a^	1.026	0.0007
Ileal scrapings						
Day 8 PI	4.66	5.42	5.06	5.29	1.059	0.513
Ileal digesta						
Day 8 PI	7.80	7.58	7.04	7.38	1.316	0.773
Colon digesta						
Day 8 PI	8.65	7.86	8.79	8.82	0.674	0.0336
Ratio lactobacilli/coliforms						
Feces						
Arrival	−0.88	−0.78	−1.51	−0.18	0.569	0.382
Day 0 PI	1.09	1.34	1.74	1.45	0.586	0.864
Day 4 PI	0.32	−1.05	−1.00	0.0007	0.598	0.201
Day 8 PI	−1.03 ^b^	−1.64 ^b^	1.44 ^a^	0.07 ^ab^	0.558	0.002
Ileal scrapings						
Day 8 PI	−0.53	0.24	−0.04	−0.88	0.582	0.449
Ileal digesta						
Day 8 PI	−0.07	0.15	−1.38	−1.16	0.707	0.222
Colon digesta						
Day 8 PI	0.49	1.17	1.27	1.56	0.811	0.759

^A^ Treatments: T1, basal diet; T2, basal diet + ZnO; T3, basal diet + plant supplement *ColiFit Icaps C*; T4, T3 + plant supplement *Phyto Ax’Cell*. ^B^ RSD: residual standard deviation. ^a,b^ values with different letters within a row indicate a significant difference at *p* ≤ 0.05.

**Table 6:** Effects of the experimental treatments on serum levels of acute-phase protein Pig-MAP and TNF-α of weaning piglets orally challenged with ETEC F4.

Parameter	Treatments ^A^				-	-
	T1	T2	T3	T4	RSD ^B^	*p*-Value
Pig-MAP ^C^	(mg/mL)	-	-	-	-	-
Day 4 PI	3.13	2.60	3.02	1.10	1.527	0.070
Day 8 PI	2.22	2.66	2.93	1.44	2.707	0.756
TNF-α ^D^	(pg/mL)	-	-	-	-	-
Day 4 PI	99.7	75.6	86.4	85.8	19.23	0.124
Day 8 PI	71.0	79.7	83.6	75.0	16.55	0.522

^A^ Treatments: T1, basal diet; T2, basal diet + ZnO; T3, basal diet + plant supplement *ColiFit Icaps C*. T4, T3 + plant supplement *Phyto Ax’Cell*. ^B^ RSD: residual standard deviation. ^C^ Pig-MAP: Major Acute-Phase Protein. ^D^ TNF-α: tumor necrosis factor α.

**Table 7:** Effects of experimental diets on ileal histomorphometry of weaning piglets orally challenged with ETEC F4.

Parameter	Treatment ^A^				-	-
	T1	T2	T3	T4	RSD ^B^	*p*-Value
Villus height (μm)	312.9	336.1	331.8	338.6	41.57	0.612
Crypt depth (μm)	214.6	179.0	191.8	184.1	34.73	0.209
Villus/crypt ratio	1.49 ^b^	1.90 ^a^	1.73 ^ab^	1.84 ^a^	0.233	0.0092
IEL^C^ (No. cel/100 μm)	6.04	5.33	5.23	4.81	1.529	0.482
GC ^D^ (No. cel/100 μm)	3.36	3.32	2.93	2.89	1.301	0.853
Mitosis ^E^ (No. cel/100 μm)	1.38	1.27	1.21	1.24	0.323	0.764

^A^ Treatments: T1, basal diet; T2, basal diet + ZnO; T3, basal diet + plant supplement *ColiFit Icaps C*; T4, T3 + plant supplement *Phyto Ax’Cell*. ^B^ RSD: residual standard deviation. ^C^ IEL= villus intraepithelial lymphocytes/100 µm. ^D^ GC= villus goblet cells/100 µm. ^E^ Mitosis = Mitosis in crypts/100 µm. ^a,b^ values with different letters within a row indicate a significant difference at *p* ≤ 0.05.

**Table 8:** Changes promoted in jejunal gene expression by the different experimental diets. Only genes with *p*-Values < 0.10 are presented. For other genes, see Table A3 (Appendix A).

Gene	Treatment	N	Mean ± SD	*p*-Value	*p*-Value FDR
*HSPA4*	T1	8	1.28 ± 0.299	0.0863	0.8765
	T2	7	1.06 ± 0.113		
	T3	6	0.96 ± 0.185		
	T4	6	1.07 ± 0.244		
*REG3G*	T1	8	1.14 ± 1.239	0.0134	0.2278
	T2	8	0.26 ± 0.443		
	T3	6	0.68 ± 0.403		
	T4	5	4.94 ± 6.053		
*SLC30A1*	T1	8	1.75 ± 0.689	<0.0001	<0.0001
	T2	8	2.84 ± 0.590		
	T3	6	1.17 ± 0.396		
	T4	6	1.11 ± 0.187		
*SLC39A4*	T1	8	0.83 ± 0.344	0.0014	0.0352
	T2	8	0.15 ± 0.049		
	T3	6	1.03 ± 0.239		
	T4	6	1.00 ± 0.236		

Values are expressed as relative fold changes normalized by housekeeping genes *GAPDH*, *ACTB*, *TBP*, and *B2M*. *p*-values are also adjusted by False Discovery Rate (FDR).

## Data Availability

The data presented in this study are available on request from the corresponding author.

## Appendix A

### Material and Methods Supplement

Detailed Description of HPLC Methodology to Analyze Active Compound in Plant Supplements

The quantification of these active compounds of *ColiFit Icaps C* was performed using High-Performance Liquid Chromatography (Shimadzu, (Kyoto, Japan), Prominence series, JPN). After an extraction in methanol and water, the solution was injected onto a C-18 column. The flow rate was maintained at 1 mL/min, and the elution made with water and acetonitrile was monitored at the 272 nm wavelength for 10 min for trans-cinnamaldehyde, the 205 nm wavelength for 10 min for eugenol, and the 205 nm wavelength during 15 min for carvacrol or thymol. The amount of each compound was calculated by external calibration of the corresponding reference standard.

The quantification of diallyl disulfide content of the garlic oil used in the mixture was made by gas chromatography coupled with a mass detector (ThermoFisher (Waltham, MA, USA), Trace GC series coupled with DSQ series, U.S.A.). The garlic oil was diluted with acetone and injected onto a TG 5MS column. The amount of diallyl disulfide content was determined by standardization, and the compound was identified using the NIST database.

The active compounds analyzed in *PhytoAx’Cell* were curcuminoids, carnosic derivatives, naringin flavonoid, salicylic derivatives, and artepillin-C. Quantification of curcuminoids content was performed by HPLC (Shimadzu, (Kyoto, Japan), Prominence series, JPN). After two extractions in methanol through a reflux condenser for 2 h, the solution was injected at a flow rate of 0.8 mL/min, and it was monitored at 425 nm wavelength using two solvents for elution: acetic acid 2% and acetonitrile. The amount of curcuminoids was calculated by external calibration of reference standard curcumin, and the result was expressed as curcumin.

Quantification of carnosic derivatives content was made using HPLC (Shimadzu (Kyoto, Japan), Prominence series, JPN). After extraction in acetone with Soxhlet and evaporation under 60 °C, the dry extract was diluted in methanol. The elution was monitored at 284 nm wavelength during 60 min using acetic acid 1%, acetonitrile and methanol as solvent for elution. The amount of carnosic derivatives was calculated by external calibration of reference standard of carvacrol and expressed as carvacrol. Quantification of naringin was performed by HPLC (Shimadzu (Kyoto, Japan), Prominence series, JPN). After an ultrasonic extraction in methanol, the solution was injected at a flow rate of 0.8 mL/min; the elution prepared with acetic acid 0.5% and acetonitrile was monitored at 283 nm wavelength for 50 min. The amount of naringin flavonoid was calculated by external calibration of reference standard of naringin and expressed as naringin. Quantification of salicylic derivatives content was made using HPLC (Agilent (Santa Clara, CA, USA), 1260 series, U.S.A.) according to the European Pharmacopoeia Monograph of Willow bark (01/2013:1583) [17]. The amount of salicylic derivatives was calculated by external calibration of reference standard of salicin and expressed as salicin.

The green propolis extract used in the mixture was analyzed separately for Artepillin-C by HPLC using Shimadzu, Prominence series, JPN. Propolis extract was diluted with 5 mL of methanol and subjected to sonication for 10 min. The solution was injected at a flow rate of 0.8 mL/L with formic acid 0.1% during 77 min, and detection was set at 275 nm. Value of Artepillin-C in *Phyto Ax’Cell* was calculated according to the extract incorporation rate.

**Table A1:** Brief description of the genes analyzed.

Gene Abbreviation	Gene Full Name	Functional Group
*OCLN*	Occludin	Intestinal barrier
*ZO1*	Zonula occludens 1	Intestinal barrier
*CLDN1*	Claudin-1	Intestinal barrier
*CLDN4*	Claudin-4	Intestinal barrier
*CLDN15*	Claudin-15	Intestinal barrier
*MUC2*	Mucin 2	Intestinal barrier
*MUC13*	Mucin 13	Intestinal barrier
*TFF3*	Trefoil factor 3	Intestinal barrier
*TLR2*	Toll-like receptor 2	Pattern recognition receptors (PRRs)
*TLR4*	Toll-like receptor 4	Pattern recognition receptors (PRRs)
*IL1β*	Interleukin 1 beta	Immune response
*lL6*	Interleukin 6	Immune response
*IL8*	Interleukin 8	Immune response
*IL10*	Interleukin 10	Immune response
*IL17A*	Interleukin 17	Immune response
*IL22*	Interleukin 22	Immune response
*IFN-γ*	Interferon gamma	Immune response
*TNF-α*	Tumor necrosis factor alpha	Immune response
*TGF-β1*	Transforming growth factor beta 1	Immune response
*CCL20*	Chemokine (C-C motif) ligand 20	Immune response
*CXCL2*	Chemokine (C-X-C motif) ligand 2	Immune response
*IFNGR1*	Interferon gamma receptor 1	Immune response
*REG3G*	Regenerating-islet derived protein 3 gamma	Immune response
*PPARGC1α*	Peroxisome proliferative activated receptor gamma, coactivator 1 alpha	Immune response
*FAXDC2*	Fatty acid hydrolase domain containing 2	Immune response
*GBP1*	Guanylate binding protein 1	Immune response
*HSPB1/HSP27*	Heat shock protein 27	Intestinal homeostasis
*HSPA4/HSP70*	Heat shock protein 70	Intestinal homeostasis
*GPX2*	Glutathione peroxidase 2	Digestive enzyme/hormone
*SOD2*	Superoxide dismutase	Digestive enzyme/hormone
*ALPI*	Intestinal alkaline phosphatase	Digestive enzyme/hormone
*SI*	Sucrase-isomaltase	Digestive enzyme/hormone
*DAO1*	Diamine oxidase	Digestive enzyme/hormone
*HNMT*	Histamine N-methyltransferase	Digestive enzyme/hormone
*ANPEP*	Aminopeptidase-N	Digestive enzyme/hormone
*IDO1*	Indoleamine 2,3-dioxygenase	Digestive enzyme/hormone
*GCG*	Glucagon	Digestive enzyme/hormone
*CCK*	Cholecystokinin	Digestive enzyme/hormone
*IGF1R*	Insulin-like growth factor 1 receptor	Digestive enzyme/hormone
*PYY*	Peptide tyrosine tyrosine	Digestive enzyme/hormone
*SLC5A1/SGLT1*	Solute carrier family 5 (sodium/glucose cotransporter) member 1	Nutrient transport
*SLC16A1/MCT1*	Monocarboxylate transporter 1	Nutrient transport
*SLC7A8*	Solute carrier family 7 (amino acid transporter light chain, L System) member 8	Nutrient transport
*SLC15A1/PEPT1*	Solute carrier family 15 (oligopeptide transporter) member 1	Nutrient transport
*SLC13A1/NAS1*	Solute carrier family 13 (sodium/sulfate symporters) member 1	Nutrient transport
*SLC11A2/DMT1*	Solute carrier family 11 (proton-coupled divalent metal ion transporter) member 2	Nutrient transport
*MT1A*	Metallothionein 1A	Nutrient transport
*SLC30A1/ZnT1*	Solute carrier family 30 (zinc transporter) member 1	Nutrient transport
*SLC39A4/ZIP4*	Solute carrier family 39 (zinc transporter) member 4	Nutrient transport
*CRHR1*	Corticotropin releasing hormone receptor 1	Stress indicators
*NR3C1/GRα*	Glucocorticoid receptor	Stress indicators
*HSD11B1*	Hydroxysteroid (11-beta) dehydrogenase 1	Stress indicators

**Table A2:** Forward and reverse primers used for gene expression analyses.

Gene	Forward Primer (5´-3´)	Reverse Primer (5´-3´)
*OCLN*	CAGGTGCACCCTCCAGATTG	CAGGCCTATAAGGAGGTGGACTT
*ZO1*	GCTATGTCCAGAATCTCGGAAAA	TGCTTCTTTCAATGCTCCATACC
*CLDN1*	CTTCGACTCCTTGCTGAATCTGA	CTTCCATGCACTTCATACACTTCAT
*CLDN4*	CCTCCGTGCTGTTCCTCAA	GAGGCACAAGCCCAGCAA
*CLDN15*	GCTATCTCCTGGTATGCCTTCAA	GGGACTTCCACACTCCTTGGT
*MUC2*	AAGGACGACACCATCTACCTCACT	GGCCAGCTCGGGAATAGAC
*MUC13*	CAGTGGAGTTGGCTGTGAAAAC	ATCAAGTTCTGTTCTTCCACATTCTTG
*TFF3*	AGAACCTGCCCGTGACCAT	CACACTGGTTCGCCGACAG
*TLR2*	CTCTCGTTGCGGCTTCCA	AAGACCCATGCTGTCCACAAA
*TLR4*	CATCCCCACATCAGTCAAGATACT	TCAATTGTCTGAATTTCACATCTGG
*IL1β*	GGTGACAACAATAATGACCTGTTATTTG	GCTCCCATTTCTCAGAGAACCA
*lL6*	CCAATCTGGGTTCAATCAGGAG	ACAGCCTCGACATTTCCCTTATT
*IL8*	GGAAAAGTGGGTGCAGAAGGT	GAGAATGGGTTTTTGCTTGTTGT
*IL10*	TGAGGCTGCGGCGCT	GAGCTTGCTAAAGGCACTCTTCA
*IL17A*	CCAGACGGCCCTCAGATTAC	ATCTTCCTTCCCTTCAGCATTG
*IL22*	TGTTCCCCAACTCTGATAGATTCC	GTTGTTCACATTTCTCTGGATATGCT
*IFN-γ*	TGACTTTGTGTTTTTCTGGCTCTT	CACTCTCCTCTTTCCAATTCTTCAA
*TNF-α*	CACCACGCTCTTCTGCCTACT	GACGGGCTTATCTGAGGTTTGA
*TGF-β1*	GCGGCAGCTCTACATTGACTT	GACCTTGCTGTACTGAGTGTCTAGG
*CCL20*	GACCATATTCTTCACCCCAGATTT	CACACACGGCTAACTTTTTCTTTG
*CXCL2*	CATGGTGAAGAAAATCATCGAGAA	GCCAGTAAGTTTCCTCCATCTCTCT
*IFNGR1*	CATGTTACCCAAATCTTTGCTGTCT	CAGTATGCACGCTTGAAATTGTC
*HSPB1/HSP27*	CGAGGAGCTGACGGTCAAG	GCAGCGTGTATTTTCGAGTGAA
*HSPA4/HSP70*	TCAATTGCCTGCGATTAATGAA	GAATGCCCCATGTCTACAAAAAC
*REG3G*	TGCCTGATGCTCCTGTCTCA	GGCATAGCAGTAGGAAGCATAGG
*PPARGC1α*	CTCTGGAACTGCAGGCCTAA	TGGAGAAGCCCTAAAAGGGTTAT
*FAXDC2*	CCATGACTACCACCATCTCAAGTT	GCAGGATCGTGTGTCTCTCGTA
*GBP1*	AGAATCCATCACAGCAGACGAGTA	CGGATACAGAGTCGAGGCAGGTTAA
*GPX2*	CAACCAATTTGGACATCAGGAG	GGGTAAAGGTGGGCTGGAAT
*SOD2*	GGGTTGGCTCGGTTTCAA	CATGCTCCCACACGTCGAT
*ALPI*	ATGTCTTCTCTTTTGGTGGCTACA	GGAGGTATATGGCTTGAGATCCA
*SI*	CGACCCCTTTTGCATGAGTT	AAGGCTGGACCCCATAGGAA
*DAO1*	GGAACCAACAGACCTTCAACTATCTC	TTCGGAATCCCAGGACCAT
*HNMT*	TGTTGAACCAAGTGCTGAACAAAT	ACTTTATGTTCTCGAGGTTTGATGTCTT
*ANPEP*	AGGGCAACGTCAAAAAGGTG	GTCAAAGCATGGGAAGGATTTC
*IDO1*	TTGGCAAATTGGAAGAAAAAGG	CCGGAAATGAGAAGAGAATATCCAT
*GCG*	AGGCGTGCCCAGGATTTT	CATCGTGACGTTTGGCAATG
*CCK*	CAGCAGGCTCGAAAAGCAC	AATCCATCCAGCCCATGTAGTC
*IGF1R*	CCGACGCGGCAACAAC	TCAGGAAGGACAAGGAGACCAA
*PYY*	CAGAGGTATGGGAAACGTGACA	CCTTCTGGCCACGACTTGAC
*SLC5A1/SGLT1*	GGCCATCTTTCTCTTACTGGCA	TCCCACTTCATGAAAAGCAAAC
*SLC16A1/MCT1*	CCTTGTTGGACCTCAGAGATTCTC	CCAGTATGTGTATTTATAGTCTCCGTATATGTC
*SLC7A8*	TGTCGCTTATGTCACTGCAATGT	GACAGGGCGACGGAAATG
*SLC15A1/PEPT1*	GGTTATCCCTTGAGCATCTTCTTC	AGTGCTCTCATTCCATAGTAGGAAAA
*SLC13A1/NAS1*	GGTACCTCCACCAACTTGATCTTC	ATCCAAAGTTGATGCAGTGACAAT
*SLC11A2/DMT1*	GTCTTTGCCGAAGCGTTTTTT	ACCACGCCCCCTTTGTAGA
*MT1A*	TGAATCCGCGTTGCTCTCT	CAGGAGCAGCAGCTCTTCTT
*SLC30A1/ZnT1*	AATTGGACCGGACAGATCCA	TCTCTGATAAGATTCCCATTCACTTG
*SLC39A4/ZIP4*	ATCTTTGGGCTCTTGCTCCTT	GCAGCCCCAGCACCTTAG
*CRHR1*	CAGGGCCCCATGATATTGG	CCGGAGTTTGGTCATGAGGAT
*NR3C1/GRα*	GGCAATACCAGGATTCAGGAACT	CCATGAGAAACATCCATGAATACTG
*HSD11B1*	GGTCAGAAGAAACTCTCAAGAAGGTG	GCGAAGGTCATGTCCTCCAT

**Table A3:** Changes promoted in jejunal gene expression by the different experimental diets.

Gene	Treatment	N	Mean ± SD	*p*-Value	*p*-Value FDR
*ALPI*	T1	8	0.87 ± 0.251	0.9159	0.9939
	T2	8	0.94 ± 0.215		
	T3	6	0.89 ± 0.374		
	T4	6	0.89 ± 0.411		
*ANPEP*	T1	7	0.90 ± 0.212	0.402	0.9514
	T2	8	0.81 ± 0.196		
	T3	6	0.86 ± 0.298		
	T4	6	0.98 ± 0.104		
*CCK*	T1	7	0.86 ± 0.268	0.8676	0.9939
	T2	8	0.88 ± 0.23		
	T3	6	0.78 ± 0.255		
	T4	6	0.86 ± 0.310		
*CCL20*	T1	7	1.83 ± 1.776	0.4469	0.9514
	T2	8	1.54 ± 0.965		
	T3	6	1.42 ± 0.384		
	T4	6	2.53 ± 1.879		
*CLDN1*	T1	7	1.32 ± 0.753	0.1767	0.8765
	T2	8	2.60 ± 2.684		
	T3	6	5.38 ± 6.288		
	T4	5	0.70 ± 0.176		
*CLDN4*	T1	8	1.13 ± 0.493	0.6631	0.9797
	T2	8	0.91 ± 0.362		
	T3	5	1.10 ± 0.629		
	T4	6	1.14 ± 0.290		
*CLDN15*	T1	8	1.01 ± 0.596	0.8469	0.9939
	T2	8	1.10 ± 0.426		
	T3	6	1.10 ± 0.465		
	T4	6	0.95 ± 0.469		
*CRHR1*	T1	7	0.93 ± 0.759	0.1459	0.8765
	T2	8	0.40 ± 0.131		
	T3	6	1.08 ± 0.770		
	T4	6	1.25 ± 1.163		
*CXCL2*	T1	8	0.81 ± 0.690	0.4778	0.9514
	T2	8	0.53 ± 0.252		
	T3	6	0.84 ± 0.373		
	T4	5	0.56 ± 0.222		
*DAO1*	T1	8	0.69 ± 0.251	0.9141	0.9972
	T2	8	0.81 ± 0.208		
	T3	6	0.76 ± 0.196		
	T4	6	0.77 ± 0.091		
*FAXDC2*	T1	8	2.28 ± 0.624	0.5783	0.9514
	T2	8	1.79 ± 0.504		
	T3	6	2.07 ± 1.026		
	T4	6	2.30 ± 0.951		
*GBP1*	T1	8	1.80 ± 0.913	0.7329	0.9797
	T2	7	2.23 ± 1.079		
	T3	6	1.85 ± 1.080		
	T4	6	1.53 ± 0.176		
*GCG*	T1	8	0.95 ± 0.278	0.4193	0.9514
	T2	8	0.86 ± 0.272		
	T3	6	0.69 ± 0.276		
	T4	6	0.95 ± 0.548		
*GPX2*	T1	8	0.63 ± 0.480	0.1116	0.8765
	T2	8	0.34 ± 0.227		
	T3	6	0.43 ± 0.372		
	T4	6	0.94 ± 0.613		
*HNMT*	T1	7	1.14 ± 0.200	0.3440	0.9514
	T2	8	1.08 ± 0.164		
	T3	6	0.99 ± 0.131		
	T4	6	0.99 ± 0.240		
*HSD11B1*	T1	8	1.11 ± 0.663	0.9682	0.9939
	T2	8	1.01 ± 0.534		
	T3	5	1.00 ± 0.729		
	T4	6	1.23 ± 1.084		
*HSPB1*	T1	8	0.65 ± 0.182	0.1040	0.8765
	T2	7	0.88 ± 0.379		
	T3	6	0.73 ± 0.235		
	T4	6	1.03 ± 0.345		
*IDO1*	T1	8	2.74 ± 2.446	0.1217	0.8765
	T2	8	3.74 ± 2.200		
	T3	5	1.45 ± 0.890		
	T4	6	1.96 ± 1.927		
*IFNGR1*	T1	8	1.33 ± 0.362	0.6421	0.9797
	T2	8	1.10 ± 0.228		
	T3	6	1.20 ± 0.520		
	T4	6	1.18 ± 0.307		
*IFNg*	T1	8	4.04 ± 2.718	0.7858	0.9797
	T2	7	2.87 ± 1.402		
	T3	6	4.01 ± 4.282		
	T4	6	1.96 ± 0.817		
*IGF1R*	T1	8	0.63 ± 0.160	0.9519	0.9939
	T2	8	0.74 ± 0.242		
	T3	6	0.80 ± 0.508		
	T4	6	0.78 ± 0.374		
*IL6*	T1	8	0.93 ± 0.651	0.8994	0.9939
	T2	8	0.82 ± 0.418		
	T3	5	1.37 ± 1.559		
	T4	6	1.05 ± 1.355		
*IL8*	T1	7	1.13 ± 0.937	0.3974	0.8446
	T2	8	0.64 ± 0.149		
	T3	6	0.90 ± 0.405		
	T4	6	1.38 ± 1.201		
*IL10*	T1	8	1.49 ± 0.735	0.2088	0.8765
	T2	8	1.35 ± 0.448		
	T3	6	1.54 ± 0.970		
	T4	6	0.94 ± 0.604		
*IL22*	T1	8	0.57 ± 0.372	0.5703	0.9514
	T2	8	0.50 ± 0.473		
	T3	6	1.32 ± 1.377		
	T4	5	0.81 ± 0.794		
*IL17A*	T1	7	0.47 ± 0.451	0.3832	0.9514
	T2	6	0.41 ± 0.375		
	T3	6	0.88 ± 0.691		
	T4	5	0.70 ± 0.597		
*IL1b*	T1	8	0.86 ± 0.299	0.5096	0.9514
	T2	7	0.70 ± 0.255		
	T3	6	1.05 ± 0.592		
	T4	6	0.78 ± 0.674		
*MUC2*	T1	8	0.94 ± 0.426	0.5725	0.9514
	T2	8	0.80 ± 0.172		
	T3	6	1.07 ± 0.183		
	T4	6	1.04 ± 0.593		
*MUC13*	T1	8	2.72 ± 1.355	0.7574	0.9797
	T2	8	2.09 ± 0.650		
	T3	6	2.64 ± 1.575		
	T4	6	2.22 ± 0.792		
*NR3C1GRa*	T1	8	1.10 ± 0.400	0.6603	0.951
	T2	8	1.21 ± 0.227		
	T3	6	1.19 ± 0.274		
	T4	6	1.06 ± 0.247		
*OCLN*	T1	8	1.74 ± 0.385	0.4075	0.9514
	T2	8	1.44 ± 0.246		
	T3	6	1.55 ± 0.532		
	T4	6	1.46 ± 0.396		
*PPARGC1a*	T1	8	1.06 ± 0.384	0.4992	0.9514
	T2	8	0.97 ± 0.215		
	T3	6	0.86 ± 0.232		
	T4	6	0.85 ± 0.217		
*PYY*	T1	8	0.97 ± 0.368	0.2386	0.8765
	T2	8	1.20 ± 0.479		
	T3	6	0.79 ± 0.218		
	T4	6	0.82 ± 0.249		
*SI*	T1	8	0.84 ± 0.249	0.1724	0.8765
	T2	8	0.66 ± 0.131		
	T3	6	0.79 ± 0.197		
	T4	6	0.64 ± 0.160		
*SLC11A2*	T1	8	1.01 ± 0.193	0.2708	0.9208
	T2	8	0.88 ± 0.207		
	T3	6	1.16 ± 0.296		
	T4	6	1.14 ± 0.425		
*SLC13A1*	T1	7	1.36 ± 0.333	0.2406	0.8765
	T2	8	1.49 ± 0.328		
	T3	6	1.19 ± 0.343		
	T4	6	1.17 ± 0.637		
*SLC15A1.PEPT1*	T1	8	1.99 ± 0.398	0.2080	0.8765
	T2	8	1.58 ± 0.337		
	T3	6	1.66 ± 0.745		
	T4	6	1.45 ± 0.414		
*SLC16A1*	T1	8	0.88 ± 0.259	0.6688	0.9797
	T2	8	0.71 ± 0.260		
	T3	6	0.90 ± 0.575		
	T4	5	0.67 ± 0.234		
*SLC5A1*	T1	8	0.97 ± 0.419	0.9530	0.9972
	T2	8	0.95 ± 0.217		
	T3	6	1.01 ± 0.363		
	T4	6	1.01 ± 0.285		
*SLC7A8*	T1	8	1.42 ± 0.883	0.9883	0.9939
	T2	8	1.29 ± 0.690		
	T3	6	1.51 ± 1.288		
	T4	6	1.38 ± 0.744		
*SOD2m*	T1	8	0.92 ± 0.281	0.7876	0.9797
	T2	8	1.05 ± 0.305		
	T3	6	0.92 ± 0.206		
	T4	6	0.97 ± 0.289		
*TFF3*	T1	8	0.90 ± 0.374	0.9939	0.9939
	T2	8	0.89 ± 0.305		
	T3	6	0.87 ± 0.143		
	T4	5	0.96 ± 0.668		
*TGFb1*	T1	8	0.94 ± 0.150	0.4796	0.9514
	T2	8	1.11 ± 0.196		
	T3	5	0.97 ± 0.188		
	T4	6	1.07 ± 0.347		
*TLR2*	T1	8	1.92 ± 1.603	0.9972	0.9972
	T2	8	1.50 ± 0.649		
	T3	5	1.28 ± 0.432		
	T4	6	2.26 ± 2.100		
*TLR4*	T1	7	0.89 ± 0.235	0.7651	0.9797
	T2	8	1.08 ± 0.310		
	T3	6	0.98 ± 0.429		
	T4	6	1.01 ± 0.634		
*TNFa*	T1	8	1.22 ± 0.418	0.7352	0.9797
	T2	8	1.12 ± 0.250		
	T3	5	0.96 ± 0.251		
	T4	6	1.20 ± 0.623		
*ZO1*	T1	8	1.14 ± 0.321	0.4949	0.9514
	T2	8	1.14 ± 0.123		
	T3	6	1.02 ± 0.278		
	T4	6	0.99 ± 0.165		

Values are expressed as relative fold changes normalized by housekeeping genes *GAPDH*, *ACTB*, *TBP*, *B2M*. *p*-Values are also adjusted by False Discovery Rate (FDR).
